# Inversion and Delayed Development of the Mandibular Left Second Premolar (Tooth 35) Associated With a Necrotic Primary Molar (Tooth 75): A Report of a Unique Pediatric Case

**DOI:** 10.7759/cureus.88399

**Published:** 2025-07-21

**Authors:** Rachid Boudi, Hind Ramdi, Majid Sakout, Houda El Khammal

**Affiliations:** 1 Department of Pediatric Dentistry, Faculty of Dental Medicine, Mohammed V University in Rabat, Rabat, MAR; 2 Department of Conservative Dentistry and Endodontics, Faculty of Dentistry, Mohammed V University in Rabat, Rabat, MAR

**Keywords:** delay development, interrelation, inverted tooth, necrosis primary molar, rare case

## Abstract

This case report details a unique clinical scenario involving a 10-year-old male patient with a severely compromised left mandibular primary second molar (tooth 75). The tooth presented with an advanced pulp-periodontal lesion and furcation involvement. Radiographic examination revealed the successor, the mandibular second premolar (tooth 35), in an inverted position and exhibiting developmental delay at Nolla Stage 6, indicating a significant delay in development.

This clinical situation highlights the interdependence between pathology in primary teeth and the normal development of their permanent successors. The report discusses diagnostic challenges, treatment planning considerations, and the importance of early intervention, with references to contemporary literature.

## Introduction

The eruption and development of permanent teeth are influenced by various local and systemic factors, including the health and position of their primary predecessors. Pulp necrosis and associated periapical pathology of primary molars can severely affect the growth and eruption trajectory of permanent successors [1,2].

The left mandibular second premolar (tooth 35) usually begins root formation around age 9, and typically reaches Nolla’s Stage 6 during this phase [3]. However, interference from infected or necrotic primary teeth may result in inversion, ectopia, or delayed calcification [4].

Inversion is a rare positional anomaly, defined as the complete axial rotation of a tooth bud, often resulting in eruption failure or ectopic positioning. Delayed dental development, particularly in a single tooth, often signals an underlying pathological condition [5]. The present case describes a rare combination of these anomalies in a child undergoing dental development, with a focus on diagnostic and therapeutic implications.

## Case presentation

**Figure 1:**
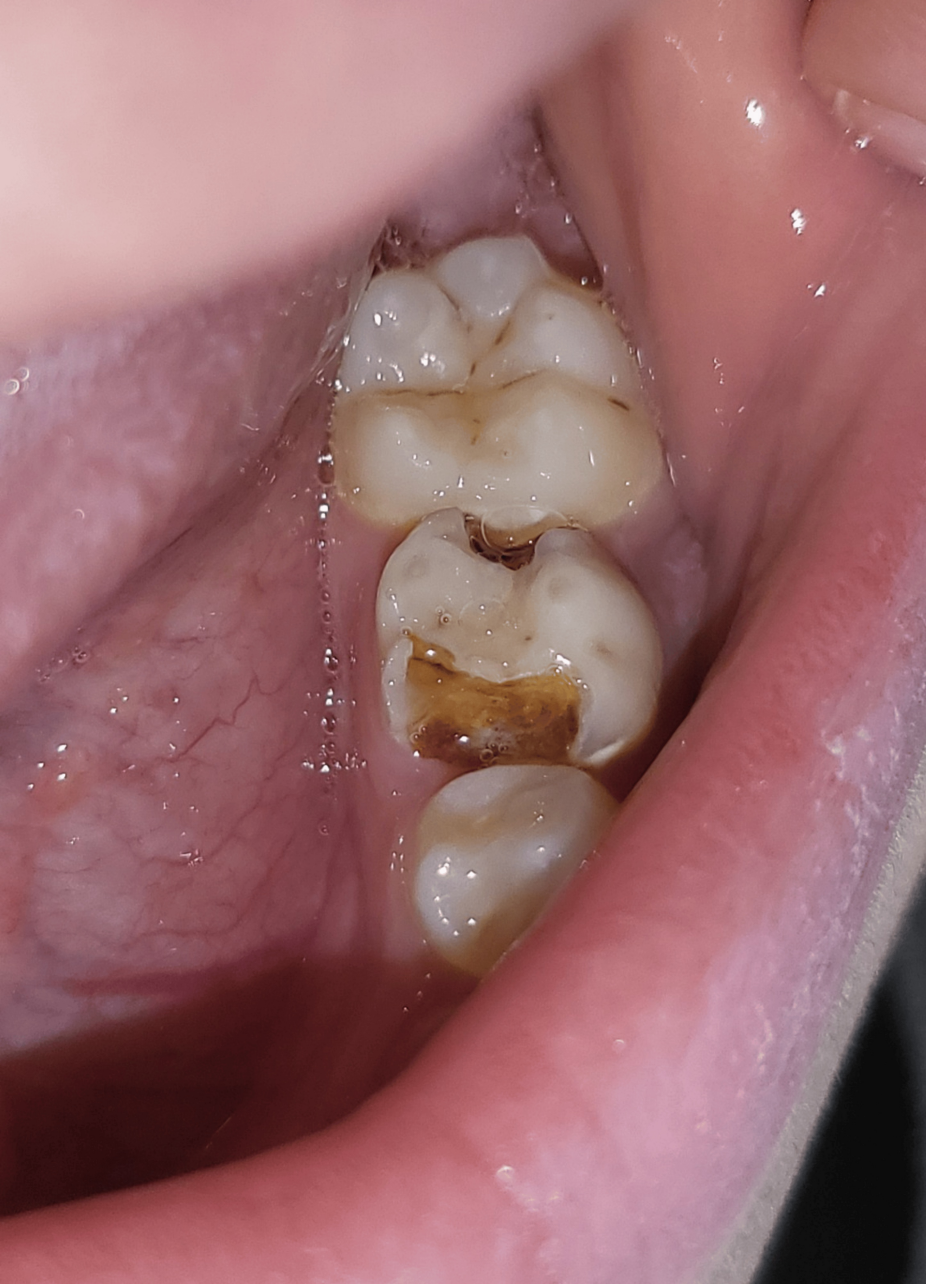
Clinical image of tooth 75 showing extensive coronal destruction with signs of infection.

**Figure 2:**
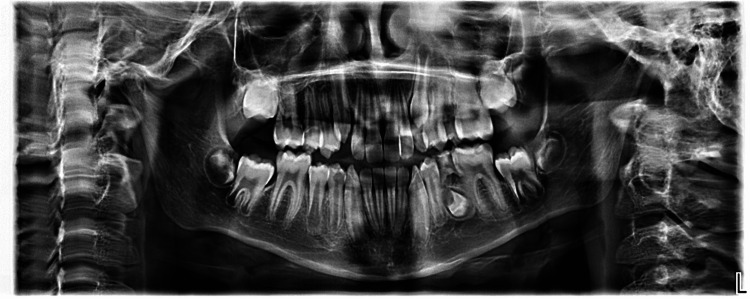
Panoramic radiograph showing inverted tooth 35 at Nolla Stage 6, with a severe furcation lesion in tooth 75.

A 10-year-old male boy was referred to the Pediatric Dental Department with complaints of pain and swelling in the lower left quadrant. The extraoral examination was within normal limits. Intraorally, the mandibular left primary second molar (tooth 75) was severely carious, with evidence of purulent discharge and buccal swelling (Figure 1). A panoramic radiograph showed a well-developed permanent dentition, except for tooth 35, which was seen in an inverted position and at Nolla Stage 6, an unusual developmental stage for the patient (Figure 2).

This stage is considered delayed for a child of this age, as tooth 35 would typically exhibit signs of root elongation by this time. Surrounding the roots of tooth 75, a distinct radiolucent area confirmed the presence of a furcation lesion, indicative of an acute pulpal-periodontal infection.

The medical and dental history reported by the parents revealed no systemic conditions, genetic disorders, or history of trauma. Considering the extensive pathology and the impact on the underlying premolar, the treatment plan involved the extraction of tooth 75, followed by long-term clinical and radiographic monitoring of tooth 35.

The management strategy was discussed in coordination with an interdisciplinary team, including orthodontists, to determine further steps if spontaneous repositioning fails. The patient is currently under regular follow-up to monitor the development of tooth 35 and to adapt treatment as needed. Informed written consent was obtained from the patient’s legal guardian for the open-access publication of this case report and the use of associated clinical images.

This case report was conducted in accordance with the ethical standards of the Institutional Research Committee and with the 1964 Helsinki Declaration and its later amendments. Written informed consent was obtained from the patient’s legal guardian for clinical examination, radiographic documentation, and publication of anonymized data and images. No identifiable personal data of the patient has been disclosed. Ethical approval was not required for this single case report, in line with institutional policy.

## Discussion

The connection between primary molar infections and the disruption in the development of permanent successors is well documented [1,2]. When a primary molar becomes necrotic, the periapical inflammation may compromise the signaling pathways between the dental follicle and the developing permanent tooth. These interactions are vital for normal tooth orientation, root formation, and the timing of eruption [3,4].

In this case, the inversion of tooth 35, detected at such an early stage of development, was highly unusual. Most cases of inversion involve impacted canines or supernumerary teeth and are often attributed to trauma or abnormal pressure within the follicular space [5,6]. Here, the absence of such contributing factors points to the possibility that chronic infection from tooth 75 influenced, locally, the dental crypt of its successor. The inflammatory environment could have led to a mechanical or biochemical disturbance, redirecting the axis of tooth 35 and delaying its progression through Nolla’s stages [3].

Nolla’s classification serves as a valuable diagnostic reference. By age 10, a mandibular second premolar is typically observed in Stage 7 or 8, with evident root elongation. A developmental standstill at Stage 6 suggests an interruption in odontogenesis [3]. Studies have shown that periapical pathology of primary molars may contribute not only to delays in eruption but also to anomalies like enamel defects, root malformations, and even tooth agenesis in extreme cases [5,6].

Management of such cases requires a carefully tailored strategy. Extraction of the necrotic tooth is essential to eliminate the source of infection. Post-extraction, periodic radiographic monitoring is crucial to assess whether spontaneous correction and root development can resume. Should spontaneous correction fail to occur, options such as orthodontic traction, surgical repositioning, or even extraction may be considered, depending on the degree of root development and available space [6,7].

The present case is unique due to the coexistence of two distinct developmental disturbances - inversion and delayed calcification - likely linked by a shared etiological factor: the necrotic status of tooth 75. This case has contributed to the growing body of evidence supporting the early and proactive management of primary tooth infections to prevent long-term sequelae in permanent dentition.

## Conclusions

This case underscores the critical impact that primary tooth pathology can have on the development of permanent successors. The simultaneous occurrence of tooth inversion and developmental delay in this patient illustrates how chronic infection in a primary molar may alter the eruption path and morphology of its successor. These findings reinforce the need for early and accurate diagnosis, interdisciplinary collaboration, and long-term follow-up in pediatric dentistry. Timely intervention is essential not only for preventing complications but also for minimizing the need for invasive procedures in the future.
